# A Mobile-Based Nutrition Tracker App Enhanced Dietitian-Guided 2:1:1 Diet-Induced Weight Loss: An 8-Week Retrospective Cohort Study in Taiwan

**DOI:** 10.3390/nu16142331

**Published:** 2024-07-19

**Authors:** Tai-Ling Chueh, Zih-Ling Wang, Yi Jing Ngu, Po-Lin Lin, Eddy Owaga, Rong-Hong Hsieh

**Affiliations:** 1 School of Nutrition and Health Sciences, College of Nutrition, Taipei Medical University, Taipei 110, Taiwan; dlchueh@tmu.edu.tw (T.-L.C.); heidi1222@tmu.edu.tw (Z.-L.W.); ma48109008@tmu.edu.tw (Y.J.N.); 2 Research Center of Nutritional Medicine, College of Nutrition, Taipei Medical University, Taipei 110, Taiwan; 3 Cofit Healthcare Inc., Taipei 104, Taiwan; berlin@cofit.me; 4 Institute of Food Bioresources Technology, Dedan Kimathi University of Technology, Nyeri 10143, Kenya; eddy.owaga@dkut.ac.ke

**Keywords:** weight loss, obesity, nutrition knowledge, mobile healthcare

## Abstract

Effective weight management interventions involve a combination of behavioral strategies focusing on dietary changes. Tracing the change through mobile apps has been proven to be a valuable platform for facilitating weight management in many countries. However, the effectiveness of mobile app-based dietary intervention on weight management in Taiwan remains to be determined. By using the designated mobile app, this study aimed to assess the efficacy of the diet intervention, which is based on a 2:1:1 portion control plate and a flexible low-carbohydrate (FLC) diet. This 8-week retrospective cohort study involved 10,297 participants who were divided into two groups: the intervention group (joined an 8-week diet intervention program with the daily diet record assessed by registered dietitians) and the control group (voluntarily using the app without instructional materials or coaching). After eight weeks of intervention, the intervention group showed a higher weight loss percentage (−4.78% vs. −1.54%), body mass index (BMI) (−1.26 kg/m^2^ vs. 0.69 kg/m^2^), and diet record completeness (73.52% vs. 28.91%) compared with the control group. With respect to gender, male participants showed higher baseline weight and higher weight loss (−6.02%) in the intervention group. In the intervention group, 2871 participants (33.4%) lost less than 4% of their weight, 5071 participants (58.9%) lost 4–8% of their body weight, and 662 participants (7.7%) lost >8% of their weight. Compared to the low-effectiveness group (weight lost <4%), the high-effectiveness group (weight lost >8%) had a significantly higher diet record completeness (91.61 ± 15.99 vs. 55.81 ± 32.92), dietary compliance (green light %) (88.93 ± 9.9 vs. 77.75 ±17.5), protein intake % (26.34 ± 2.85 vs. 23.49 ± 3.56), and fat intake % (49.66 ± 6.36 vs. 44.05 ± 7.37). Most importantly, the high-effectiveness group had a lower carbohydrate intake % (24.1 ± 7.86 vs. 32.46 ± 9.61). The results remained significant after being stratified by gender. This study found that the use of online applications plus the intervention of dietitians is beneficial for short-term weight loss. The composition of nutrients and dietary compliance also significantly impacted weight loss.

## 1. Introduction

Obesity is a global public health concern. According to the National Health and Nutrition Survey in Taiwan (NAHSIT), it has been observed that from 1975 to 2016, the prevalence of obesity among men has increased from 3% to 11%, and from 6% to 15% among women [1]. Obesity is a risk factor for many chronic diseases, including diabetes, cardiovascular diseases, and cancer. Further, it leads to an increased mortality rate from comorbidities [2]. Therefore, it is crucial to effectively control the continued increase in overweight and obesity prevalence. The fundamental strategy to improve obesity is lifestyle modification [3], such as adjusting dietary patterns and increasing physical activity. These approaches take precedence over pharmaceutical treatments or surgical interventions [4].

However, implementing these intervention measures in resource-limited environments can be challenging.

Weight loss clinics and weight management centers often require individuals to attend in person, demanding significant patience and determination for the effective results [5]. Emergencies or missed appointments can also reduce the effectiveness of weight loss and decrease self-confidence during the weight loss journey. Additionally, physiological, environmental, and behavioral factors make it challenging to maintain long-term weight loss, especially without structured support [6].

Fortunately, with the development of technology, mobile healthcare has become a trend due to its advantages, including shortening the distance and improving the communication between patients and medical personnel, aiding in disease management and providing real-time feedback, thus offering long-term value in healthcare services [7]. Many studies have proven that using mHealth applications is widely accepted as a weight management tool and is effective for promoting weight loss among overweight and obese individuals [8,9]. Previous research has shown that clinically significant weight loss outcomes are associated with the number of articles read in the short and long term, recorded weight, step counts, exercise frequency, and messages sent to coaches, as well as dietary intake and the number of meals recorded per week [10].

Standard obesity treatments include dietary self-monitoring (DSM), which involves documenting the details of one’s dietary intake, including calorie amounts and the timing of consumption. Paper-based DSM is a standard behavioral correction method in clinical settings, and higher self-monitoring adherence is related to weight loss. Previous studies have shown that more frequent self-monitoring of dietary intake, along with improved consistency and completeness of self-monitoring are associated with better weight loss outcomes [11,12]. However, as the duration of weight loss extends, long-term (over one year) adherence to dietary self-monitoring decreases, leading to a decrease in weight loss effectiveness [13]. In contrast, the weight management programs that provide dedicated coaches to support participants have demonstrated higher levels of engagement and greater success in achieving weight loss goals [14,15].

In addition to the widespread use of mHealth apps, various dietary strategies for weight management are well-known interventions [16,17]. One approach using “food-based” whole dietary patterns and the portion control plate model seemed more effective than examining nutrients in isolation [18,19]. The 211 dietary principle is a concept based on this approach, dividing food intake for each meal into a 2:1:1 ratio, comprising vegetables, complex carbohydrates, and proteins with low saturated fat, to prevent excessive carbohydrate consumption [20].

While studies of app-based health coaching interventions have demonstrated positive results on weight management in many countries [14,21,22], there is lack of evidence regarding the effectiveness of online coaching programs for weight loss in Taiwan. The purpose of this study was to investigate the weight loss effectiveness achieved by using portion-controlled plates and the 211 dietary principle, managed by a dedicated dietitian through a mobile app.

## 2. Materials and Methods

### 2.1. Study Design

This retrospective study used data from Cofit Healthcare Inc., Taipei, Taiwan. All the data were collected from the COFIT application, which facilitates daily diet records to achieve self-monitoring weight management. All members voluntarily use the app, with some enrolled to participate in an 8-week structured weight management program guided by dedicated registered dietitians. The study received approval from the Institutional Review Board of Taipei Medical University (N202302024).

Data were collected from app users who agreed to provide their personal information by signing the registered user agreement. The study included Taiwanese adults aged 18–65 years who had no missing data in body measurements or dietary records. Participants were categorized into two groups based on their involvement in the program: the intervention group, consisting of individuals enrolled in the structured intervention program, and the control group, composed of those who were not specifically recruited for the study and did not receive any instructional materials or coaching from dietitians. They used the app for self-monitoring their diet and weight on a self-directed, voluntary basis.

### 2.2. COFIT Application

COFIT is a mobile application developed by Cofit Healthcare Inc. in Taiwan. It offers personalized programs for weight loss, muscle gain, and glucose monitoring. The app includes a comprehensive Asian food database, body composition tracking, community support, and access to a dietitian for personalized coaching. Users upload their daily food consumption, water intake, and exercise content. If users agree to join the associated program, they are matched with a dedicated dietitian to receive a personalized nutrition plan.

### 2.3. Intervention Group

The intervention program in this study is called the flexible low-carbohydrate (FLC) program. This intervention includes personalized dietary advice and online nutrition guidance based on the 211 dietary principle. The 211 diet refers to a portion control plate where the ratio of vegetable, protein and complex carbohydrate is 2:1:1, respectively. This fits the concept of a low carbohydrate diet (LCD), which involves consuming less than 130 g carbohydrates (CHO) per day, with a nutrient distribution of less than 30% from CHO, 20–30% from protein, and 50–60% from fat [23,24]. The low CHO approach is considered a viable option for reversing metabolic-associated chronic diseases and combating the obesity epidemic [25].

The program lasted for 8 weeks and each participant was matched with a dedicated registered dietitian. During the program, participants received a nutritional consultation and were asked to upload their daily diet records. They received the results of their daily nutrition intake and personalized recommendation evaluated by the dietitians.

### 2.4. Control Group

The control group consisted of members who voluntarily used the mobile app function to log diet and weight data. The mobile app healthcare provider did not provide any instructional materials or coaching throughout the study.

### 2.5. Measurements

The primary outcome was a change in body weight over two months. Participants were asked to upload their body weight using the app daily. The secondary outcomes include macronutrient intakes, diet record completeness, and dietary compliance.

#### 2.5.1. Diet Record Completeness

To assess participants’ adherence to maintaining comprehensive dietary intake records, diet record completeness was defined as the ratio of days with uploaded entries to the total duration of the program. This criterion accommodated varying meal frequencies, ranging from one to multiple meals per day.

#### 2.5.2. Dietary Compliance

Dietary choices were assessed using a traffic light system where food items were categorized as green (best choices), yellow (choose carefully), or red (limit) [26,27,28]. This approach enabled the evaluation of participants’ adherence to the recommend dietary guidelines throughout the intervention period.

#### 2.5.3. Macronutrient Intakes

Registered dietitians analyzed diet record photos uploaded by participants to calculate total daily caloric intake and the percentage distribution of carbohydrates, proteins, and fats. This method provided detailed insights into participants’ nutritional intake patterns.

### 2.6. Statistical Analysis

Descriptive statistics were utilized to provide an overview of the baseline characteristics and changes in weight, BMI, fat mass, and waist circumference among the participants at week 0, week 4, and week 8. These measures were presented as the means and standard deviations (mean ± SD). Furthermore, participants were stratified into three subgroups based on their weight loss performance, using predetermined thresholds: low (<4% weight loss), moderate (4% to 8% weight loss), or high (>8% weight loss). To assess the effectiveness of weight loss, independent two-sample, two-sided *t*-tests were employed, comparing the intervention group with the control group at weeks 0, 4, and 8. Additionally, stratification analyses were conducted using an analysis of variance (ANOVA) to investigate the relationship between adherence to diet record completeness goals and weight loss outcomes. This exploration encompassed multiple factors, such as the number of photo uploads, total calorie intake, carbohydrate intake rate, protein intake rate, and fat intake rate, allowing for a comprehensive examination of adherence to diet record completeness goals and its impact on weight loss outcomes. To mitigate the potential for multiple comparisons, Bonferroni’s adjustment was implemented. All statistical analyses were performed using R software (version 4.2.1).

## 3. Results

### 3.1. Baseline Characteristics

Among 10,297 adults who were enrolled in this study, 8604 individuals completed the 8-week diet intervention program (intervention group). The remaining 1693 individuals voluntarily used the app to record weight and diet data without instructional materials or coaching (control group). The majority of participants were female (7853/8604, 91.3% intervention vs. 1257/1693, 74.2% control). The mean age was 35.2 ± 7.5 and 33.7 ± 9.9 years, respectively, in two groups (intervention vs. control). In the intervention group, some participants had common metabolic comorbidities, including type 2 diabetes (1.8%), hypertension (5%), hyperlipidemia (4.3%), and non-alcoholic fatty liver disease (NAFLD) (15.1%). Most participants had a college degree (undergraduate) (56.6%) and worked indoors (71.5%) (Table 1).

### 3.2. Changes in Weight Loss

Table 2 shows the change in weight and BMI at weeks 0, 4, and 8 during the 8-week FLC program. Both the intervention and control groups demonstrated a clear trend in weight loss throughout the program (*p*-trend < 0.001). The intervention group showed significant weight loss from week 0 to week 8 (*p*-trend <0.001, *p* < 0.001) in all subgroups (total, female and male). On the other hand, the control group only showed significant difference in weight loss in the total and female subgroups (*p* value = 0.02 and 0.005, respectively). The total mean weight loss percentage for the intervention group was 4.97%, which is significantly higher than the control group (1.67%). Similar results were seen in the weight loss percentages for female and male, with a decrease of 4.63% in the female intervention group and 6.24% in the male intervention group compared to 2.09% in the female control group and 0.65% in the male control group. For BMI, the intervention group decreased from 26.1 ± 4.4 to 24.9 ± 4.2 kg/m^2^ (*p* < 0.001), while the control group only reported minimal changes with no statistical difference.

### 3.3. Program Adherence and Weight Loss Effectiveness

As shown in Table 3, participants were categorized into three groups based on the achieved weight loss percentage: high effectiveness (>8% weight loss), moderate effectiveness (4–8%), and low effectiveness (<4%). Among the participants, 33% lost <4% of their weight, 59% lost 4–8%, and 8% lost >8% during the program. Participants with higher weight loss percentages tended to have higher baseline weights and BMIs compared to those with lower weight loss percentages. Furthermore, when considering gender differences, the results showed that, on average, male participants had higher baseline weights and BMIs than female participants. However, male participants also achieved more significant weight loss than female participants.

The three factors used as indicators to evaluate app engagement and adherence to the intervention program included macronutrient intake, diet record completeness, and dietary compliance. Among females, participants with a higher effectiveness in weight loss had a significantly higher calorie intake (*p*-trend <0.01) but lower carbohydrate consumption (*p*-trend < 0.001). While total calorie consumption among males showed no significant difference among the three groups, those in the high-effectiveness group (>8% weight loss) had a lower carbohydrate intake, and higher protein and fat intake (*p*-trend < 0.001).

Both females and males in the high-effectiveness group had a significantly higher diet record completeness (91.61 ± 15.99% vs. 55.81 ± 32.92%, *p* < 0.001) and higher green light percentage, which indicates higher dietary compliance (88.93 ± 9.9% vs. 77.75 ± 17.5%, *p* < 0.001).

## 4. Discussion

This study found that an 8-week mobile app-based FLC program effectively reduced weight in overweight and obese adults. The weight loss percentage in the intervention group was significantly greater than the control group. Three indicators (macronutrient intake, diet record completeness, and dietary compliance) were shown to be relevant to weight loss effectiveness.

Our results showed that a reduction in carbohydrate intake is more influential than calorie restriction on weight loss. This finding aligns with previous studies, which revealed that an LCD resulted in greater weight loss and BMI reduction than a low-calorie diet [29,30]. Furthermore, Sun et al. reported that an LCD alone, without a calorie restriction, is sufficient to improve body weight compared to a calorie-restricted diet over a 12-week intervention [31]. In our study, we also found that male participants with different weight loss effectiveness showed no significant difference in their total calorie intake, but those with higher effectiveness had a significantly lower carbohydrate intake. One possibility is that an LCD requires more total energy expenditure (TEE) than a high-carbohydrate diet [32]. Previous studies revealed that in trials lasting more than 2.5 weeks, when the carbohydrate intake decreased by 10% of energy intake (EI), TEE increased by 50 kcal/day [33]. An LCD often involves an increased consumption of proteins (20–30% of EI). It has been well established that proteins have a higher thermic effect (20–30%) compared to carbohydrates (5–10%) and fats (0–3%). Thus, a greater intake of protein is a potential contributor to higher TEE in an LCD [34,35].

From the results of the intervention group compared with the control group, it can be seen that, in addition to using the app, an intervention with structured guidance by a dedicated professional dietitian has a better effect on weight loss. One possibility is that most people prefer individualized treatment which is adapted to the patient’s individual and psychological needs [36]. Additionally, the knowledge sharing and guided support from the dietitian might be extra motivating factors for participants enrolled in the program to improve their daily dietary practices compared to those in the control group [37]. Allen et al. investigated 68 obese people receiving smartphone intervention plus counseling or not. The result demonstrated that the participants in the intensive counseling plus self-monitoring smartphone group tended to lose more weight than those only using the smartphone app [38].

The results of the present study also revealed that although males had a higher baseline body weight and BMI, they achieved more noticeable weight loss and reduction in BMI than females over 8 weeks. Our findings are consistent with previous studies. Christensen et al. conducted an 8-week study of 2224 individuals to investigate metabolic outcomes after a specific diet intervention in males and females. The results showed that females lost twice as much fat-free mass as males, leading to a reduction in the basal metabolic rate (BMR) and potentially less weight loss in females compared to males. Additionally, males lost significantly more body weight than females and showed a larger reduction in metabolic syndrome Z-score, proving the gender-specific differences in weight loss between the two genders [39]. Aeronica et al. examined 12-month changes in body weight and composition by comparing a low-carbohydrate (LC) to a low-fat diet (LF) in both genders. The results indicated that after adjusting for adherence, males experienced greater weight loss than females in the LC group [40]. Physically, males can lose more weight through dietary interventions than females due to their higher muscle mass, higher basal metabolic rate (BMR), and total energy expenditure. A meta-analysis by Williams et al. found that males might lose more weight than females in diet plus exercise interventions [41]. Susanto et al. also indicated the importance of examining weight change for males and females separately to prevent masking potential differences and assist with better weight loss outcomes [42].

Adherence to the weight management smartphone application is considered as a predictor of short-term weight loss [3]. In our study, adherence to the program was examined based on uploaded diet record completeness and dietary compliance. The results demonstrated that greater adherence to dietary records was associated with an increased effectiveness in weight loss. Participants who achieved higher effectiveness exhibited higher completeness in their dietary records and a higher percentage of “green light” ratings. Behavioral interventions have been proven to be an effective therapeutic strategy for weight loss maintenance. When individuals fully agree with behavioral goals and these goals facilitate autonomous motivation, their efforts are most likely to lead to sustained behavioral change [43]. Jacobs et al. discovered that the users of the Noom app, which is similar to the COFIT app in our study, attained a significantly lower BMI after three months if they had higher adherence [3]. Hauser et al. found that participants with a higher level of adherence to the assigned diet and higher quality diet (according to HEI-2010 score) were most successful in reducing their BMI [44,45]. Oh et al. also encouraged active participation to maximize the effectiveness of the mHealth app [4].

## 5. Conclusions

This study evaluates the effectiveness of a mobile app-based dietary intervention on weight management in Taiwan, focusing on portion control and a flexible low-carbohydrate (FLC) diet. Significant findings emerged from an 8-week retrospective cohort study involving 10,297 participants. The intervention group, which received structured dietary guidance and daily diet record assessments by registered dietitians, demonstrated notable improvements in weight loss percentage and BMI reduction compared to those of the control group that used the mHealth application without personal coaching. Our data consolidate the association between dedicated guidance programs and successful weight loss. The secondary result of this study demonstrates that a greater adherence to professional guidance can lead to better weight loss effectiveness. These findings reveal the importance of adherence to nutritional recommendations in achieving meaningful weight management outcomes. Overall, this study highlights the value of online applications coupled with personalized dietary interventions facilitated by dietitians in promoting short-term weight loss. Future research could explore the long-term sustainability of these interventions and examine additional factors that influence dietary adherence and weight management success in diverse populations. These insights can help improve strategies for combating obesity and promoting healthier lifestyles.

## Figures and Tables

**Table 1:** Baseline characteristics (*n* = 10,297).

	Intervention Group			Control Group			*p*-Value ᵃ	*p*-Value ᵇ	*p*-Value ᶜ
	Female	Male	Total	Female	Male	Total			
***n*(%)**	7853 (91.3)	751 (8.7)	8604 (100)	1257 (74.2)	436 (25.8)	1693 (100)			
Age (yrs)	35.3 ± 7.4	34.9 ± 8.6	35.2 ± 7.5	34.1 ± 9.9	32.5 ± 10.1	33.7 ± 9.9	<0.001	<0.001	<0.001
<20 (n,%)	31 (0.4)	9 (1.2)	40 (0.5)	40 (3.2)	16 (3.7)	56 (3.3)			
20–29 (n,%)	1641 (20.9)	188 (25)	1829 (21.3)	432 (34.4)	185 (42.4)	617 (36.4)			
30–39 (n,%)	4172 (53.1)	376 (50.1)	4548 (52.9)	435 (34.6)	136 (31.2)	571 (33.7)			
40–49 (n,%)	1697 (21.6)	136 (18.1)	1833 (21.3)	238 (18.9)	63 (14.4)	301 (17.8)			
50–59 (n,%)	265 (3.4)	26 (3.5)	291 (3.4)	103 (8.2)	31 (7.1)	134 (7.9)			
<60 (n,%)	46 (0.6)	14 (1.9)	60 (0.7)	9 (0.7)	5 (1.1)	14 (0.8)			
Weight (kg)	66.9 ± 10.8	83.3 ± 16.3	68.4 ± 12.3	62.2 ± 11.7	77.2 ± 14.9	66 ± 14.2	<0.001	<0.001	<0.001
BMI (kg/m^2^)	25.9 ± 4.2	28.9 ± 4.7	26.1 ± 4.3	24.2 ± 4.2	26.4 ± 4.6	24.6 ± 4.3	<0.001	<0.001	<0.001
**Disease History (*n*,%)**									
Diabetes	141 (1.8)	12 (1.6)	153 (1.8)	N/A	N/A	N/A	N/A	N/A	N/A
Hypertension	340 (4.3)	90 (12)	430 (5)	N/A	N/A	N/A	N/A	N/A	N/A
Hyperlipidemia	299 (3.8)	74 (9.9)	373 (4.3)	N/A	N/A	N/A	N/A	N/A	N/A
Nonalcoholic fatty liver disease (NAFLD)	1119 (14.2)	179 (23.8)	1298 (15.1)	N/A	N/A	N/A	N/A	N/A	N/A
**Education (*n*,%)**									
Graduated	1946 (24.8)	204 (27.2)	2150 (25)	N/A	N/A	N/A	N/A	N/A	N/A
Undergraduate	4470 (56.9)	398 (53)	4868 (56.6)	N/A	N/A	N/A	N/A	N/A	N/A
High school	311 (4)	35 (3.3)	346 (4)	N/A	N/A	N/A	N/A	N/A	N/A
**Occupation (*n*,%)**									
Indoor	5607 (71.4%)	543 (72.3%)	6150 (71.5%)	N/A	N/A	N/A	N/A	N/A	N/A
Outdoor	364 (4.6%)	74 (9.9%)	438 (5.1%)	N/A	N/A	N/A	N/A	N/A	N/A
Night shift	381 (4.9%)	52 (6.9%)	433 (5%)	N/A	N/A	N/A	N/A	N/A	N/A
Households	1501 (19.1%)	82 (10.9%)	1583 (18.4%)	N/A	N/A	N/A	N/A	N/A	N/A

Continuous data are presented as the mean ± SD, while categorical data are presented as number (*n*) and percentage. The *p*-value was derived using the Student *t*-test. *p* < 0.05: indicates statistically significant differences. ^a^: *p*-value for comparison between the total control group and total intervention group. ^b^: *p*-value between the female control group and female intervention group. ^c^: *p*-value between the male control group and male intervention group. Abbreviation: N/A = not applicable.

**Table 2:** Change in weight and BMI at weeks 0, 4, and 8 during the two months of the FLC program.

	Intervention Group (*n* = 8604)			*p*-Value ᵃ	*p*-Value ᵇ	*p*-Value ᶜ	*p*-Trend	Weight Loss (%)	Control Group (*n* = 1693)			*p*-Value ᵃ	*p*-Value ᵇ	*p*-Value ᶜ	*p*-Trend	Weight Loss (%)
	Week 0	Week 4	Week 8						Week 0	Week 4	Week 8					
**Weight** (**kg**)																
Total	68.4 ± 12.3	66.2 ± 12	65 ± 11.4	<0.001	<0.001	<0.001	<0.001	−4.97% *	66 ± 14.2	65.4 ± 14	64.9 ± 14	0.22	0.3	0.02	<0.001	−1.67%
Female	66.9 ± 10.8	64.9 ± 10	63.8 ± 10.2	<0.001	<0.001	<0.001	<0.001	−4.63% *	62.2 ± 11.7	61.4 ± 12	60.9 ± 11.4	0.09	0.28	0.005	<0.001	−2.09%
Male	83.3 ± 16.3	79.8 ± 15	78.1 ± 14.5	<0.001	0.03	<0.001	<0.001	−6.24% *	77.2 ± 14.9	76.9 ± 15	76.7 ± 14.3	0.77	0.84	0.61	<0.001	−0.65%
**BMI** (**kg/m^2^**)																
Total	26.1 ± 4.4	25.2 ± 4	24.9 ± 4.2	<0.001	<0.001	<0.001	<0.001	N/A	24.6 ± 4.3	24.3 ± 4	25.2 ± 37.7	0.04	0.33	0.52	0.4382	N/A
Female	26 ± 4.3	25 ± 4	24.8 ± 4.1	<0.001	0.002	<0.001	<0.001	N/A	24.2 ± 4.2	23.9 ± 4	25 ± 41.7	0.07	0.35	0.5	0.4193	N/A
Male	27.6 ± 5	28 ± 4	25.9 ± 4.5	0.09	<0.001	<0.001	<0.001	N/A	26.4 ± 4.6	26.4 ± 4	26.2 ± 4.4	>0.99	0.48	0.51	<0.001	N/A

Continuous data are presented as the mean ± SD, while categorical data are presented as number (*n*) and percentage. The *p*-trend value was derived using a one-way ANOVA to evaluate the trend among data points across different time points (Week 0, Week 4, and Week 8). ^a^: *p*-value for comparison between week 0 and week 4. ^b^: *p*-value for comparison between week 4 and week 8. ^c^: *p*-value for comparison between week 8 and week 0. Weight loss (%) was calculated as follows: [(weight loss % week 8 − weight loss % week 0)/weight loss % week 0] ×100. *: significant difference in weight loss % between the intervention and control groups, *p*-value < 0.001. Abbreviation: N/A = not applicable.

**Table 3:** Anthropometry indexes and diet record in the intervention group stratified by gender and weight loss effectiveness.

	Female			*p*-Trend	Male			*p*-Trend	Total			*p*-Trend
Weight Loss Percentage	>8% (*n* = 501)	4–8% (*n* = 4633)	<4% (*n* = 2719)		>8% (*n* = 161)	4–8% (*n* = 438)	<4% (*n* = 152)		>8% (*n* = 662)	4–8% (*n* = 5071)	<4% (*n* = 2871)	
Age (yrs)	35.98 ± 6.55	35.25 ± 7.3	35.23 ± 7.61	0.1408	33.7 ± 7.32	35.18 ± 8.05	35.14 ± 11.04	0.006122	35.42 ± 6.81	35.24 ± 7.36	35.22 ± 7.83	0.8111
Baseline weight (kg)	69.11 ± 12.41	67.56 ± 10.22	65.48 ± 11.31	<0.001	93.95 ± 16.38	80.49 ± 14.04	79.88 ± 17.4	<0.001	75.15 ± 17.18	68.68 ± 11.21	66.24 ± 12.14	<0.001
Baseline BMI (kg/m^2^)	26.62 ± 4.37	26.04 ± 4.42	25.73 ± 4.18	<0.001	30.04 ± 4.92	26.98 ± 4.48	26.62 ± 5.56	<0.001	27.45 ± 4.74	26.12 ± 4.44	25.78 ± 4.27	<0.001
**Effectiveness**												
∆Weight (kg)	6.53 ± 1.74	3.65 ± 0.91	1.67 ± 0.97	<0.001	9.49 ± 3.47	4.64 ± 1.31	2.11 ± 1.11	<0.001	7.25 ± 2.61	3.74 ± 0.99	1.69 ± 0.98	<0.001
∆Weight (%)	9.43 ± 1.4	5.4 ± 0.97	2.53 ± 1.4	<0.001	9.96 ± 2.01	5.73 ± 1.06	2.66 ± 1.42	<0.001	9.56 ± 1.58	5.42 ± 0.98	2.54 ± 1.41	<0.001
∆BMI (kg/m^2^)	2.52 ± 0.64	1.41 ± 0.37	0.65 ± 0.38	<0.001	3.02 ± 1.03	1.55 ± 0.43	0.7 ± 0.38	<0.001	2.64 ± 0.78	1.42 ± 0.37	0.65 ± 0.38	<0.001
**Macronutrient Intake**												
Calorie (kcal)	1125.8 ± 159.2	1114.9 ± 171.5	1104.7 ± 192.9	<0.01	1302.5 ± 269.7	1327.9 ± 248.6	1289 ± 312.2	0.726	1168.6 ± 206.1	1133.1 ± 188.9	1113.9 ± 204.5	<0.001
Carbohydrate (%)	23.09 ± 6.93	27.5 ± 9.09	32.44 ± 9.55	<0.001	26.83 ± 9.73	28.74 ± 9.75	32.81 ± 10.59	<0.001	24.1 ± 7.86	27.61 ± 9.16	32.46 ± 9.61	<0.001
Protein (%)	26.53 ± 2.56	25.06 ± 3.09	23.5 ± 3.54	<0.001	25.76 ± 3.57	25.03 ± 3.56	23.37 ± 3.95	<0.001	26.34 ± 2.85	25.05 ± 3.14	23.49 ± 3.56	<0.001
Fat (%)	50.38 ± 5.78	47.45 ± 7.02	44.06 ± 7.32	<0.001	47.42 ± 7.49	46.22 ± 7.16	43.82 ± 8.25	<0.001	49.66 ± 6.36	47.34 ± 7.04	44.05 ± 7.37	<0.001
**Diet Record**												
Diet record Completeness (%)	95.26 ± 10.49	81.61 ± 22.04	56.21 ± 32.78	<0.001	80.27 ± 23.27	76.75 ± 25.75	48.7 ± 34.64	<0.001	91.61 ± 15.99	81.19 ± 22.43	55.81 ± 32.92	<0.001
Light green (%)	90.51 ± 8.03	85.1 ± 11.65	77.94 ± 17.36	<0.001	84.01 ± 13.08	82.89 ± 12.74	74.4 ± 19.62	<0.001	88.93 ± 9.9	84.91 ± 11.77	77.75 ± 17.5	<0.001
Light yellow (%)	7.6 ± 6.22	12.1 ± 9.61	17.75 ± 14.64	<0.001	12.68 ± 10.29	13.91 ± 10.82	20.86 ± 17.48	<0.001	8.84 ± 7.72	12.26 ± 9.74	17.91 ± 14.81	<0.001
Light red (%)	1.89 ± 3.07	2.8 ± 4.1	4.31 ± 7.14	<0.001	3.32 ± 4.87	3.19 ± 4.92	4.74 ± 8.01	0.09005	2.24 ± 3.64	2.83 ± 4.18	4.33 ± 7.19	<0.001

Continuous data are presented as the mean ± SD, while categorical data are presented as number (*n*) and percentage. The *p*-trend value was derived using a one-way ANOVA to evaluate the trend among data points across different effectiveness (>8%, 4–8%, <4%).

## Data Availability

The original contributions presented in the study are included in the article, further inquiries can be directed to the corresponding author.

## Abbreviations

FLC	flexible low-carbohydrate
BMI	body mass index
DSM	dietary self-monitoring
NAFLD	nonalcoholic fatty liver disease
TEF	thermic effect of food
LCD	low-carbohydrate diet
BMR	basal metabolic rate
MetS	metabolic syndrome

## References

[1] Hung T.S., Fox K.R. (2022). Prevalence of Overweight and Obesity in Taiwanese Adults: Estimates from National Surveys, 1990–2017. J. Med. Health.

[2] Chin S.O., Keum C., Woo J., Park J., Choi H.J., Woo J.T., Rhee S.Y. (2016). Successful weight reduction and maintenance by using a smartphone application in those with overweight and obesity. Sci. Rep..

[3] Jacobs S., Radnitz C., Hildebrandt T. (2017). Adherence as a predictor of weight loss in a commonly used smartphone application. Obes. Res. Clin. Pract..

[4] Oh B., Yi G.H., Han M.K., Kim J.S., Lee C.H., Cho B., Kang H.C. (2018). Importance of Active Participation in Obesity Management Through Mobile Health Care Programs: Substudy of a Randomized Controlled Trial. JMIR mHealth uHealth.

[5] Beratarrechea A., Lee A.G., Willner J.M., Jahangir E., Ciapponi A., Rubinstein A. (2014). The impact of mobile health interventions on chronic disease outcomes in developing countries: A systematic review. Telemed. J. e-Health.

[6] May C.N., Cox-Martin M., Ho A.S., McCallum M., Chan C., Blessing K., Behr H., Blanco P., Mitchell E.S., Michaelides A. (2023). Weight loss maintenance after a digital commercial behavior change program (Noom Weight): Observational cross-sectional survey study. Obes. Sci. Pract..

[7] Sharma S., Kumari B., Ali A., Yadav R.K., Sharma A.K., Hajela K., Singh G.K. (2022). Mobile technology: A tool for healthcare and a boon in pandemic. J. Fam. Med. Prim. Care.

[8] Kim E.K., Kwak S.H., Jung H.S., Koo B.K., Moon M.K., Lim S., Jang H.C., Park K.S., Cho Y.M. (2019). The Effect of a Smartphone-Based, Patient-Centered Diabetes Care System in Patients with Type 2 Diabetes: A Randomized, Controlled Trial for 24 Weeks. Diabetes Care.

[9] Mao A.Y., Chen C., Magana C., Barajas K.C., Olayiwola J.N. (2017). A Mobile Phone-Based Health Coaching Intervention for Weight Loss and Blood Pressure Reduction in a National Payer Population: A Retrospective Study. JMIR mHealth uHealth.

[10] Carey A., Yang Q., DeLuca L., Toro-Ramos T., Kim Y., Michaelides A. (2021). The Relationship Between Weight Loss Outcomes and Engagement in a Mobile Behavioral Change Intervention: Retrospective Analysis. JMIR mHealth uHealth.

[11] Wadden T.A., Tronieri J.S., Butryn M.L. (2020). Lifestyle modification approaches for adult obesity. Am. Psychol..

[12] Patel M.L., Wakayama L.N., Bennett G.G. (2021). Self-Monitoring via Digital Health in Weight Loss Interventions: A Systematic Review among Adults with Overweight or Obesity. Obesity.

[13] Peterson N.D., Middleton K.R., Nackers L.M., Medina K.E., Milsom V.A., Perri M.G. (2014). Dietary self-monitoring and long-term success with weight management. Obesity.

[14] Painter S.L., Ahmed R., Kushner R.F., Hill J.O., Lindquist R., Brunning S., Margulies A. (2018). Expert Coaching in Weight Loss: Retrospective Analysis. J. Med. Internet Res..

[15] Van Wier M.F., Ariëns G.A., Dekkers J.C., Hendriksen I.J., Smid T., Van Mechelen W. (2009). Phone and e-mail counselling are effective for weight management in an overweight working population: A randomized controlled trial. BMC Public Health.

[16] Kim J.Y. (2021). Optimal Diet Strategies for Weight Loss and Weight Loss Maintenance. J. Obes. Metab. Syndr..

[17] Makris A., Foster G.D. (2011). Dietary approaches to the treatment of obesity. Psychiatr. Clin. N. Am..

[18] Tapsell L.C., Neale E.P., Satija A., Hu F.B. (2016). Foods, Nutrients, and Dietary Patterns: Interconnections and Implications for Dietary Guidelines. Adv. Nutr..

[19] Florentino R.F., Tee E., Hardinsyah R., Ismail M.N., Suthutvoravut U., Hop L.T. (2016). Food-Based Dietary Guidelines of Southeast Asian Countries: Part 2—Analysis of Pictorial Food Guides. Malays. J. Nutr..

[20] Ha K., Nam K., Song Y. (2020). A moderate-carbohydrate diet with plant protein is inversely associated with cardiovascular risk factors: The Korea National Health and Nutrition Examination Survey 2013–2017. Nutr. J..

[21] Carter M.C., Burley V.J., Nykjaer C., Cade J.E. (2013). Adherence to a smartphone application for weight loss compared to website and paper diary: A pilot randomized controlled trial. J. Med. Internet Res..

[22] Spring B., Duncan J.M., Janke E.A., Kozak A.T., McFadden H.G., DeMott A., Pictor A., Epstein L.H., Siddique J., Pellegrini C.A. (2013). Integrating technology into standard weight loss treatment: A randomized controlled trial. JAMA Intern. Med..

[23] Bilsborough S.A., Crowe T.C. (2003). Low-carbohydrate diets: What arethe potential short- and long-term health implications?. Asia Pac. J. Clin. Nutr..

[24] Adam-Perrot A., Clifton P., Brouns F. (2006). Low-carbohydrate diets: Nutritional and physiological aspects. Obes. Rev..

[25] Hite A.H., Berkowitz V.G., Berkowitz K. (2011). Low-Carbohydrate Diet Review. Nutr. Clin. Pract..

[26] Brown R.D. (2011). The traffic light diet can lower risk for obesity and diabetes. NASN Sch. Nurse.

[27] Epstein L.H. (2022). A Brief History and Future of the Traffic Light Diet. Curr. Dev. Nutr..

[28] Vasconcelos C., Almeida A., Cabral M., Ramos E., Mendes R. (2019). The Impact of a Community-Based Food Education Program on Nutrition-Related Knowledge in Middle-Aged and Older Patients with Type 2 Diabetes: Results of a Pilot Randomized Controlled Trial. Int. J. Environ. Res. Public Health.

[29] Gardner C.D., Kiazand A., Alhassan S., Kim S., Stafford R.S., Balise R.R., Kraemer H.C., King A.C. (2007). Comparison of the Atkins, Zone, Ornish, and LEARN Diets for Change in Weight and Related Risk Factors among Overweight Premenopausal Women: The A TO Z Weight Loss Study: A Randomized Trial. JAMA.

[30] Bazzano L.A., Hu T., Reynolds K., Yao L., Bunol C., Liu Y., Chen C.S., Klag M.J., Whelton P.K., He J. (2014). Effects of Low-Carbohydrate and Low-Fat Diets: A Randomized Trial. Ann. Intern. Med..

[31] Sun J., Ruan Y., Xu N., Wu P., Lin N., Yuan K., An S., Kang P., Li S., Huang Q. (2023). The effect of dietary carbohydrate and calorie restriction on weight and metabolic health in overweight/obese individuals: A multi-center randomized controlled trial. BMC Med..

[32] Ebbeling C.B., Bielak L., Lakin P.R., Klein G.L., Wong J.M.W., Luoto P.K., Wong W.W., Ludwig D.S. (2020). Energy Requirement Is Higher during Weight-Loss Maintenance in Adults Consuming a Low- Compared with High-Carbohydrate Diet. J. Nutr..

[33] Ludwig D.S., Dickinson S.L., Henschel B., Ebbeling C.B., Allison D.B. (2021). Do Lower-Carbohydrate Diets Increase Total Energy Expenditure? An Updated and Reanalyzed Meta-Analysis of 29 Controlled-Feeding Studies. J. Nutr..

[34] Calcagno M., Kahleova H., Alwarith J., Burgess N.N., Flores R.A., Busta M.L., Barnard N.D. (2019). The Thermic Effect of Food: A 367 Review. J. Am. Coll. Nutr..

[35] Pesta D.H., Samuel V.T. (2014). A high-protein diet for reducing body fat: Mechanisms and possible caveats. Nutr. Metab..

[36] Endevelt R., Gesser-Edelsburg A. (2014). A qualitative study of adherence to nutritional treatment: Perspectives of patients and dietitians. Patient Prefer. Adherence.

[37] Dempsey K., Mottola M.F., Atkinson S.A. (2023). Comparative Assessment of Diet Quality and Adherence to a Structured Nutrition and Exercise Intervention Compared with Usual Care in Pregnancy in a Randomized Trial. Curr. Dev. Nutr..

[38] Allen J.K., Stephens J., Dennison Himmelfarb C.R., Stewart K.J., Hauck S. (2013). Randomized controlled pilot study testing use of smartphone technology for obesity treatment. J. Obes..

[39] Christensen P., Meinert Larsen T., Westerterp-Plantenga M., Macdonald I., Martinez J.A., Handjiev S., Poppitt S., Hansen S., Ritz C., Astrup A. (2018). Men and women respond differently to rapid weight loss: Metabolic outcomes of a multi-centre intervention study after a low-energy diet in 2500 overweight, individuals with pre-diabetes (PREVIEW). Diabetes Obes. Metab..

[40] Aronica L., Rigdon J., Offringa L.C., Stefanick M.L., Gardner C.D. (2021). Examining differences between overweight women and men in 12-month weight loss study comparing healthy low-carbohydrate vs. low-fat diets. Int. J. Obes..

[41] (2015). Williams, R.L.; Wood, L.G.; Collins, C.E.; Callister, R. Effectiveness of weight loss interventions—Is there a difference between men and women: A systematic review. Obes. Rev..

[42] Susanto A., Burk J., Hocking S., Markovic T., Gill T. (2022). Differences in weight loss outcomes for males and females on a low-carbohydrate diet: A systematic review. Obes. Res. Clin. Pract..

[43] Flore G., Preti A., Carta M.G., Deledda A., Fosci M., Nardi A.E., Loviselli A., Velluzzi F. (2022). Weight Maintenance after Dietary Weight Loss: Systematic Review and Meta-Analysis on the Effectiveness of Behavioural Intensive Intervention. Nutrients.

[44] Guenther P.M., Kirkpatrick S.I., Reedy J., Krebs-Smith S.M., Buckman D.W., Dodd K.W., Casavale K.O., Carroll R.J. (2014). The Healthy Eating Index-2010 is a valid and reliable measure of diet quality according to the 2010 Dietary Guidelines for Americans. J. Nutr..

[45] Hauser M.E., Hartle J.C., Landry M.J., Fielding-Singh P., Shih C.W., Rigdon J., Gardner C.D. (2024). Association of dietary adherence and dietary quality with weight loss success among those following low-carbohydrate and low-fat diets: A secondary analysis of the DIETFITS randomized clinical trial. Am. J. Clin. Nutr..

